# Integrative transcriptomic and experimental analyses prioritize TPT1 as a PANoptosis-associated candidate molecular marker in sarcopenia

**DOI:** 10.3389/fcell.2026.1912393

**Published:** 2026-08-20

**Authors:** Shijie Dong, Min Wang, Chen Liang, Peiyu Xu, Ziyi Ye, Jingqing Yao, Zhongquan Tang, Ting Ou, Xiaomin Zhao, Xinyu Dai, Yuntao Li, Guozhong Ji

**Affiliations:** 1 Department of Neurology, The Second Affiliated Hospital of Nanjing Medical University, Nanjing, China; 2 Department of General Practice, The Second Affiliated Hospital of Nanjing Medical University, Nanjing, China

**Keywords:** candidate molecular marker, machine learning, PANoptosis, sarcopenia, single-nucleus RNA sequencing, skeletal-muscle aging, TPT1

## Abstract

**Background:**

Sarcopenia lacks sensitive molecular markers for early detection, and its relationship with integrated inflammatory cell-death programs remains unclear. PANoptosis integrates apoptotic, pyroptotic, and necroptotic signaling and therefore provides a plausible framework for investigating inflammatory-stress remodeling in aging skeletal muscle.

**Methods:**

We integrated four bulk-transcriptomic datasets from the Gene Expression Omnibus into a training cohort (66 controls; 37 sarcopenia) and used GSE111016 as an external validation cohort (20 controls; 20 sarcopenia). We intersected differentially expressed genes with a curated PANoptosis-associated gene set and then performed enrichment analysis; least absolute shrinkage and selection operator (LASSO), random forest and extreme gradient boosting (XGBoost) feature selection; nomogram and receiver operating characteristic (ROC) analyses; CIBERSORT immune-cell deconvolution; and single-nucleus RNA sequencing (snRNA-seq) reanalysis. We assessed tumor protein, translationally controlled 1 (TPT1) expression in D-galactose-treated mouse and C2C12 models.

**Results:**

Among 608 differentially expressed genes, 47 overlapped with the curated PANoptosis-associated gene set. These genes were enriched in apoptotic signaling; cytokine, nuclear factor kappa B (NF-κB), tumor necrosis factor (TNF), and nucleotide-binding oligomerization domain (NOD)-like receptor pathways; regulated necrosis; extracellular-matrix remodeling; and impaired oxidative phosphorylation. Three machine-learning algorithms converged on neurotrophic receptor tyrosine kinase 1 (NTRK1), TPT1, and TNF receptor-associated protein 1 (TRAP1). TPT1 showed the strongest single-gene discrimination, with areas under the ROC curve of 0.819 (95% confidence interval [CI], 0.737–0.900) in the training cohort and 0.753 (95% CI, 0.598–0.907) in the external cohort. Immune-cell deconvolution linked the candidate genes to estimated mast-cell, plasma-cell, cluster of differentiation 8-positive (CD8^+^) T-cell, and macrophage proportions. Single-nucleus analysis of 97,154 nuclei from 17 donors showed broad TPT1 expression across myonuclear, satellite-cell, stromal, endothelial, and immune compartments, with lower expression in older muscle. Network and gene set variation analyses associated lower TPT1 expression with inflammatory, oxidative-stress, cell-death, and stress-adaptive pathways. In D-galactose-treated mice and C2C12 myotubes, muscle-wasting or senescence-like changes coincided with lower TPT1 protein abundance.

**Conclusion:**

This study prioritizes TPT1 as a candidate molecular marker associated with the bulk-transcriptomic sarcopenia phenotype. The aging-muscle and D-galactose analyses provide biological context but do not establish sarcopenia specificity or causality. Prospective clinical validation and functional perturbation studies are required.

## 1 Introduction

Sarcopenia is an age-related disease characterized by the progressive loss of skeletal-muscle mass and function (Sayer et al., 2024). It contributes to frailty, functional decline, and increased mortality in older adults (Beaudart et al., 2025). Its reported prevalence depends substantially on the diagnostic definition applied (Petermann-Rocha et al., 2022). Its pathogenesis remains incompletely understood and involves chronic inflammation, physical inactivity, impaired protein synthesis, mitochondrial dysfunction, and endocrine alterations (Sayer et al., 2024; Zuo et al., 2025). Sarcopenia can lead to physical disability and loss of independence, yet effective clinical treatments remain limited, imposing substantial burdens on patients, families, and healthcare systems.

Recent sarcopenia guidelines emphasize early identification and timely intervention (Chen et al., 2020; Cruz-Jentoft et al., 2019). Diagnosis currently combines three domains: muscle mass, evaluated by dual-energy X-ray absorptiometry (DXA) or bioelectrical impedance analysis (BIA); muscle strength, commonly measured by handgrip dynamometry; and physical performance, assessed by gait speed or the chair-stand test. Although clinically useful, these measures may be insufficiently sensitive to early biological changes. Reliable molecular markers could therefore complement existing assessments and support earlier screening and intervention (Liu et al., 2025).

Cellular senescence and inflammaging provide an additional biological link between aging and muscle decline. Senescent myogenic, stromal, vascular, and immune cells can accumulate in aged skeletal muscle and produce a senescence-associated secretory phenotype comprising cytokines, chemokines, growth factors, and matrix-remodeling mediators. This chronic secretory state can impair muscle-stem-cell function, disrupt regenerative niches, and reinforce local and systemic low-grade inflammation. Recent single-cell and multi-omic studies have identified heterogeneous senescent populations and pro-inflammatory niche interactions in aging muscle, while emphasizing that senescence depends on cellular and tissue context rather than representing a uniform state (Liang et al., 2022; Zhang et al., 2022; Moiseeva et al., 2023; Lai et al., 2024; Li et al., 2025).

Sarcopenia progression is linked to several cell-death and stress-response pathways, including autophagy, pyroptosis, apoptosis, and ferroptosis (Xie et al., 2023; Ru et al., 2025; Wu et al., 2023). PANoptosis integrates key features of apoptosis, pyroptosis, and necroptosis through PANoptosome assembly, indicating that these pathways can interact rather than operate in isolation under pathological stress (Pandian and Kanneganti, 2022; Gao et al., 2024; Pandeya and Kanneganti, 2024; Zhu et al., 2023). A recent study used PANoptosis-associated genes to identify and experimentally examine candidate sarcopenia biomarkers (Deng et al., 2025). Nevertheless, cross-cohort robustness, single-nucleus cellular context, and the boundaries of computational inference remain insufficiently resolved.

To address these gaps, we combined integrative transcriptomics, machine-learning screening, immune-cell deconvolution, human single-nucleus RNA sequencing (snRNA-seq) reanalysis, and experimental expression assessment to characterize PANoptosis-related molecular alterations in sarcopenia. We prioritized neurotrophic receptor tyrosine kinase 1 (NTRK1), tumor protein, translationally controlled 1 (TPT1), and TNF receptor-associated protein 1 (TRAP1), of which TPT1 showed the most consistent discrimination across the training and external cohorts. We then examined the cellular context and experimental expression pattern of TPT1 while treating regulatory networks, pathway scores, and compound associations explicitly as hypothesis-generating analyses.

## 2 Materials and methods

### 2.1 Data collection

We retrieved bulk skeletal-muscle transcriptomic data from the Gene Expression Omnibus (GEO). GSE111006 and GSE111010 were integrated with GSE238215 and GSE226151 into a training cohort comprising 66 control samples and 37 samples from individuals with sarcopenia; GSE111016 served as an independent external cohort comprising 20 control samples and 20 samples from individuals with sarcopenia (Migliavacca et al., 2019; Kim et al., 2024; Zuo et al., 2025). Diagnostic criteria varied among cohorts. We included only samples that met the diagnostic thresholds specified for their respective datasets, thereby maintaining clearly defined sarcopenia and control groups for subsequent analyses. Supplementary Table S1 provides the biopsy site, diagnostic definition, age, sex, and sample-selection criteria for each dataset.

We operationally defined PANoptosis as a coordinated molecular framework encompassing apoptosis, necroptosis, and pyroptosis. To construct a reproducible pathway-based candidate set, we retrieved human gene sets from the Molecular Signatures Database (MSigDB; release v2024.1. Hs) (Subramanian et al., 2005). Gene Ontology Biological Process (GOBP) terms were based on the Gene Ontology (GO) resource (The Gene Ontology Consortium, 2019), and Kyoto Encyclopedia of Genes and Genomes (KEGG) terms were based on KEGG (Kanehisa et al., 2016). Apoptosis-related genes were obtained from GOBP_INTRINSIC_APOPTOTIC_SIGNALING_PATHWAY (GO:0097193), GOBP_EXTRINSIC_APOPTOTIC_SIGNALING_PATHWAY (GO:0097191), GOBP_EXECUTION_PHASE_OF_APOPTOSIS (GO:0097194), KEGG_APOPTOSIS (hsa04210), and REACTOME_APOPTOSIS (R-HSA-109581). Necroptosis and regulated-necrosis genes were obtained from GOBP_NECROPTOTIC_PROCESS (GO:0070266) and REACTOME_REGULATED_NECROSIS (R-HSA-5218859), whereas pyroptosis-related genes were obtained from GOBP_PYROPTOSIS (GO:0070269) and REACTOME_PYROPTOSIS (R-HSA-5620971). After merging these sets and removing duplicates, we obtained 781 unique PANoptosis-related genes (PRGs). Supplementary Table S2 lists the standardized symbols, full gene names, and assigned cell-death categories.

**FIGURE 1:**
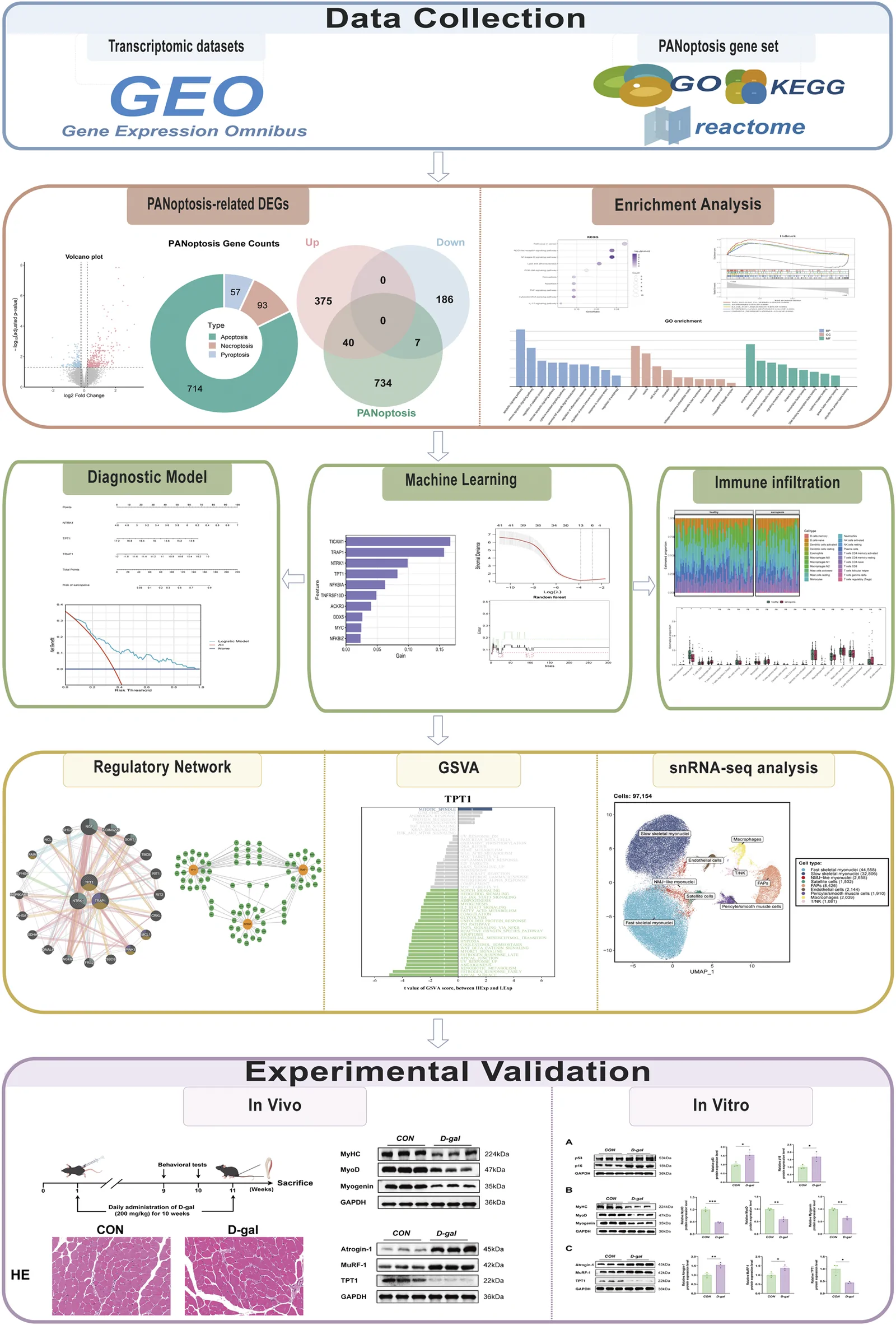
Overall study design. Four Gene Expression Omnibus (GEO) bulk skeletal-muscle transcriptomic datasets were integrated as the training cohort (n = 103; control samples, n = 66; sarcopenia samples, n = 37), and GSE111016 served as the external validation cohort (n = 40; control samples, n = 20; sarcopenia samples, n = 20). A PANoptosis-associated search set comprising 781 unique genes was compiled from Gene Ontology (GO), Kyoto Encyclopedia of Genes and Genomes (KEGG), and Reactome gene sets and intersected with differentially expressed genes. Functional enrichment, machine-learning feature selection, exploratory model evaluation, CIBERSORT immune-cell deconvolution, regulatory-network prediction, and gene set variation analysis (GSVA) were subsequently performed. Single-nucleus RNA sequencing data provided aging-related cellular context using 97,154 nuclei retained after quality control from 17 donors (young, n = 6; older, n = 11). TPT1 expression was then examined in D-galactose-treated mouse and C2C12 models.

We obtained snRNA-seq data from GEO accession GSE167186, which contains muscle-biopsy samples from 6 young and 11 older individuals and 143,051 nuclei before quality control (Perez et al., 2022). Because this dataset contrasts age groups rather than clinically defined sarcopenia and control groups, we used it only to provide aging-related cellular context. Figure 1 summarizes the study workflow.

### 2.2 Data preprocessing and identification of differentially expressed PANoptosis-related genes

For integrative analysis of GSE111010, GSE111006, GSE238215, and GSE226151, we retained genes present in all four datasets and merged the raw count matrices by official gene symbol. We adjusted dataset-associated batch effects with ComBat-seq from the sva R package, using study identifier as the batch variable and disease status as a biological covariate (Zhang et al., 2020). We then used DESeq2 to analyze differential expression in the integrated training cohort (Love et al., 2014). We defined differentially expressed genes (DEGs) by an adjusted P value <0.05 and an absolute log2 fold change (|log2FC|) > 0.25. This threshold was selected to capture modest but potentially relevant transcriptional changes while controlling false-positive findings. We intersected the DEGs with the curated PRGs to obtain differentially expressed PANoptosis-related genes (DE-PRGs) for subsequent analysis.

### 2.3 Functional enrichment analysis

We investigated the functions and pathway associations of the DE-PRGs by performing GO enrichment analysis and KEGG pathway analysis with the clusterProfiler R package (Yu et al., 2012). We also performed gene set enrichment analysis (GSEA) to examine coordinated pathway-level differences (Subramanian et al., 2005), using an adjusted P value <0.05 as the significance threshold.

### 2.4 Machine learning–based screening of candidate genes

We restricted feature selection to the training cohort. We fitted a random forest (RF) model with 300 trees, ranked gene importance by mean decrease in Gini impurity, and used the out-of-bag (OOB) error as an internal estimate of classification performance. We fitted least absolute shrinkage and selection operator (LASSO) logistic regression (alpha = 1) with 10-fold stratified cross-validation and selected the penalty parameter with the conservative lambda.1se criterion, defined as the largest λ within one standard error of the minimum cross-validated deviance. We trained extreme gradient boosting (XGBoost) models with five-fold stratified cross-validation and early stopping after 20 rounds without improvement. The cross-validated area under the ROC curve (AUC; eval_metric = “auc”) determined the optimal number of boosting iterations, and gain determined feature importance (booster = “gbtree”, objective = “binary:logistic”, eta = 0.05, max_depth = 3, subsample = 0.8, colsample_bytree = 0.8, lambda = 1). All cross-validation folds were stratified by binary outcome to preserve the proportions of control and sarcopenia samples (Breiman, 2001; Tibshirani, 1996; Chen and Guestrin, 2016).

To limit overfitting and improve robustness, we derived a candidate-gene set from each algorithm. LASSO retained genes with nonzero coefficients at lambda.1se, whereas RF and XGBoost each contributed the 10 highest-ranked genes according to mean decrease in Gini impurity and gain, respectively. Genes selected by all three algorithms were designated consensus candidate genes for subsequent validation and model construction. We performed no feature selection in the external cohort (GSE111016), which was reserved exclusively for independent evaluation of expression direction and discriminative performance. We quantified discrimination by the AUC and estimated 95% confidence intervals (CIs) with DeLong’s method (DeLong et al., 1988). Analyses used R version 4.4.3 and the glmnet, randomForest, xgboost, and pROC packages.

### 2.5 Nomogram construction and receiver operating characteristic analysis

We constructed a nomogram from the three consensus candidate genes with the rms R package to estimate sample-level group-membership probability. We assessed agreement between predicted and observed probabilities with calibration curves. Decision-curve analysis (DCA) compared the model with treat-all and treat-none strategies across the displayed threshold-probability range (Vickers and Elkin, 2006). ROC-curve analysis evaluated the discrimination of each candidate gene and the nomogram, with the AUC used as the summary measure.

### 2.6 Immune-cell deconvolution

To characterize the immune-cell composition associated with sarcopenia, we applied CIBERSORT with the LM22 signature matrix and 1,000 permutations to estimate the relative proportions of 22 immune-cell types (Newman et al., 2015; Chen et al., 2018). Stacked bar plots summarized sample-level estimates, and boxplots displayed between-group differences. We calculated Spearman rank correlations between candidate-gene expression and estimated immune-cell proportions and visualized the results with ggplot2. Because deconvolution estimates depend on the reference signature, normalization, and tissue composition, we treated all results as exploratory (Avila Cobos et al., 2020).

### 2.7 Single-nucleus RNA sequencing analysis

We processed the snRNA-seq data with Seurat version 5.0.1 (Hao et al., 2024). For each donor, quality control removed nuclei whose log1p total counts or log1p detected-gene counts lay more than three median absolute deviations (MADs) above or below the median. We also removed nuclei with a mitochondrial-read percentage more than three MADs above the donor-specific median or above an absolute threshold of 20%. Scrublet identified putative doublets, which we excluded (Wolock et al., 2019). We normalized the filtered data with NormalizeData and selected 2,000 highly variable genes with FindVariableFeatures. We performed principal component analysis (PCA) after ScaleData and RunPCA and selected the first 20 principal components from the elbow plot. FindNeighbors and FindClusters generated graph-based clusters at a resolution of 0.5, which we visualized with Uniform Manifold Approximation and Projection (UMAP). We manually annotated clusters using canonical skeletal-muscle markers. To generate a 50-gene PANoptosis-related module score at single-nucleus resolution, we retrieved the 50 highest-ranked core PANoptosis-related genes from GeneCards (Stelzer et al., 2016; Supplementary Table S3) and applied SeuratAddModuleScore to normalized expression values. This score summarizes aggregate expression of the selected gene set. Because the source dataset compares young and older muscle rather than sarcopenia and control samples, we interpreted all findings only as age-associated cellular context.

### 2.8 Chromosomal distribution, GeneMANIA interaction network, and single-gene gene set variation analysis

We visualized the chromosomal positions of the candidate genes with RCircos version 1.2.2 (Zhang et al., 2013). We submitted TPT1, TRAP1, and NTRK1 to GeneMANIA (https://genemania.org/) to examine their predicted functional-interaction landscape and retained the 20 genes with the highest functional-similarity scores (Warde-Farley et al., 2010). We then used gene set variation analysis (GSVA) to estimate sample-level activity scores for the 50 Hallmark pathways with the GSVA R package (Hänzelmann et al., 2013); Hallmark gene sets were obtained from MSigDB (Subramanian et al., 2005). For each candidate gene, we divided samples at the median expression value and compared pathway scores between the high- and low-expression groups with Welch’s t-test. We treated pathways with |t| > 1 and nominal P < 0.05 as exploratory associations.

### 2.9 Regulatory-network construction and Drug Signatures Database compound-signature analysis

We used NetworkAnalyst to construct microRNA (miRNA)–gene and transcription factor (TF)–gene networks, drawing miRNA–target interactions from TarBase and TF–target interactions from JASPAR (Zhou et al., 2019). We also queried the Drug Signatures Database (DSigDB) through Enrichr to identify compound or perturbagen signatures associated with the candidate-gene set (Yoo et al., 2015). We interpreted these database-derived associations as hypothesis-generating.

### 2.10 Animal experiments

We purchased 12 eight-week-old male C57BL/6 mice from Beijing Vital River Laboratory Animal Technology Co., Ltd. Mice were housed at 23 °C ± 2 °C and 50%–60% relative humidity under a 12-h light/dark cycle, with food and water available *ad libitum*. Experiments began at 9 weeks of age after a 1-week acclimation period. We randomly allocated mice to D-galactose or vehicle groups (n = 6 biological replicates per group). The D-galactose group received a daily intraperitoneal injection of D-galactose (200 mg/kg) for 10 weeks, whereas controls received an equal volume of phosphate-buffered saline (PBS). We recorded body weight weekly and monitored food intake, hair loss, frailty, and locomotor activity. The Animal Ethics Committee of Nanjing Medical University approved the study (Approval No. IACUC-2601012), which followed the relevant institutional guidelines.

### 2.11 Behavioral tests

We measured forelimb grip strength with a grip-strength meter (XR501, Shanghai Xin Ruan, China). For each trial, we placed the mouse on the apparatus and pulled the grip bar horizontally away from the animal until it released its grip. Each mouse completed three consecutive trials separated by 5-min rest intervals. We averaged the three measurements and normalized the result to body weight.

We used the inverted-grid test to assess limb-muscle strength and endurance. The apparatus consisted of a 30 × 30 cm wire-mesh grid positioned 20 cm above an open surface (Deacon, 2013), with a soft foam pad placed underneath to prevent injury. After placing a mouse on the grid, we inverted the grid and recorded the latency to fall. Each mouse completed three trials separated by 10-min rest intervals, and the longest latency was used for analysis.

We assessed muscular endurance with a motorized treadmill (BW-TDM709, Shanghai Ruanlong, China). Mice were acclimated to the treadmill for 5 days. During the test, the belt ran at 10 m/min for 60 s and then increased by 3 m/min every 3 min, with each speed transition lasting 10 s. Exhaustion was defined as failure to resume running despite repeated gentle prodding with a soft probe or remaining on the shock grid for 10 s.

We completed all behavioral tests 1–2 weeks before tissue collection to minimize acute exercise effects. The investigator performing these tests was blinded to group allocation, and each mouse represented one independent biological replicate.

### 2.12 Tissue collection

Immediately after euthanasia, we dissected the gastrocnemius, tibialis anterior, and quadriceps muscles from both hindlimbs. We recorded wet muscle weights and normalized them to body weight. We fixed the left gastrocnemius in 4% paraformaldehyde for histological analysis and immediately froze the remaining muscles in liquid nitrogen. Samples were stored at −80 °C until analysis.

### 2.13 Hematoxylin and eosin staining

We fixed gastrocnemius samples in 4% paraformaldehyde (PFA) for 24 h, embedded them in paraffin, cut 5-μm sections, and stained the sections with hematoxylin and eosin (H&E). We scanned the sections with a Panoramic MIDI system and quantified mean myofiber cross-sectional area (CSA) with Image-Pro Plus.

### 2.14 Cell culture

Mouse myoblast C2C12 cells were obtained from the Cell Bank of the Chinese Academy of Sciences (Shanghai, China) and cultured in DMEM (Gibco, United States) supplemented with 10% fetal bovine serum (FBS) (Gibco, United States). All cells were cultured with 1% penicillin/streptomycin (Gibco, United States) at 37 °C in 5% CO_2_. Cells were passaged when they reached 60%–70% confluence, and passages beyond ten were not used. To induce myogenic differentiation, C2C12 myoblasts at 90%–100% confluence were washed with PBS, and the medium was switched to differentiation medium (DM) consisting of DMEM supplemented with 2% horse serum (Gibco, United States). DM was changed daily for 5 days until fully differentiated myotubes were formed. To induce myotube atrophy, D-galactose (20 g/L) was added to the differentiation medium, and the cells were cultured for an additional 48 h (Guo et al., 2024; Ye et al., 2024).

### 2.15 Western blotting

The appropriate quantity of RIPA lysis buffer (1% PMSF, 1% PIC) was added to the samples of gastrocnemius tissue and C2C12 myotubes. The samples were then subjected to a crushing and centrifugation process, resulting in the separation of the supernatant. The supernatant was then boiled with a 1% SDS sample buffer. The proteins were then separated on SDS-PAGE and electro-transferred to polyvinylidene fluoride (PVDF) membranes. Following a 1 h incubation period in milk, the membranes were exposed to primary antibodies at 4 °C overnight. This was followed by a 1 h incubation period with secondary antibodies at room temperature. After washing with TBST buffer, the immunocomplexes were then visualized using Tanon™ High Signal ECL Protein Blotting Substrate (Tanon, Tanon, China) and an automated digital gel/chemiluminescence image analysis system (4600 S F, Tanon, China). The detailed antibodies used in this study are listed in Supplementary Table S4.

### 2.16 Statistical analysis

Animal phenotypic, functional, and histological outcomes used six biological replicates per group; all western blots used three biological replicates per group or condition. Data are presented as the mean ± SD, with individual biological-replicate values shown where applicable. For two-group comparisons, we first assessed normality with the Shapiro–Wilk test. When both groups met the normality criterion (P > 0.05), we assessed homogeneity of variance with an F test and used an unpaired two-tailed Student’s t-test for equal variances or Welch’s t-test for unequal variances. When either group violated the normality assumption, we used the Mann–Whitney U test. All tests were two-sided, and P < 0.05 indicated statistical significance. Analyses used R version 4.4.3 and GraphPad Prism version 9.0.

## 3 Results

### 3.1 Identification of differentially expressed PANoptosis-related genes

**FIGURE 2:**
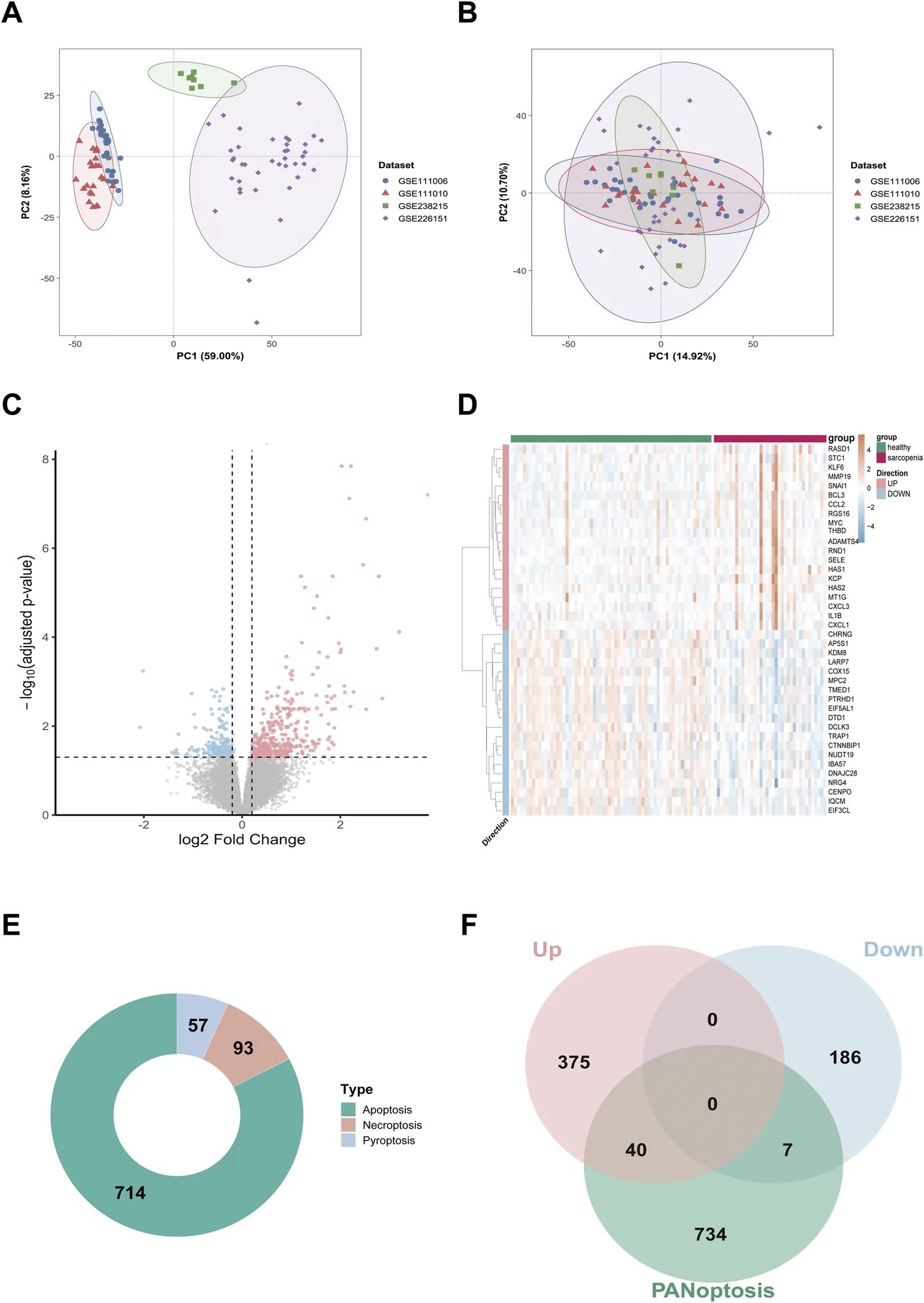
Identification of differentially expressed PANoptosis-associated genes in sarcopenia. Analyses used the integrated training cohort (n = 103; control samples, n = 66; sarcopenia samples, n = 37) **(A,B)** Principal component analysis before **(A)** and after **(B)** batch-effect correction. Each point represents one sample; point color and shape denote source dataset **(C)** Volcano plot of differential expression. Pink, blue, and gray points denote upregulated, downregulated, and nonsignificant genes, respectively; significance was defined as adjusted P < 0.05 and |log2 fold change| > 0.25. A total of 415 genes were upregulated and 193 were downregulated **(D)** Heatmap of the 20 most upregulated and 20 most downregulated genes. The upper annotation identifies control and sarcopenia samples, and the blue-to-orange scale represents row-scaled expression **(E)** Apoptosis-, necroptosis-, and pyroptosis-associated category counts before cross-category duplicate removal (714, 93, and 57 genes, respectively). These categories were not mutually exclusive and yielded 781 unique genes after symbol harmonization and duplicate removal **(F)** Venn diagram showing overlap of the 415 upregulated genes, 193 downregulated genes, and 781 PANoptosis-associated genes; 40 upregulated and 7 downregulated genes overlapped the curated set.

To characterize sarcopenia-associated transcriptional features, we integrated four GEO datasets (GSE111006, GSE111010, GSE226151, and GSE238215) and adjusted dataset-associated batch effects with ComBat-seq. PCA and boxplots showed that the pronounced dataset-driven separation observed before correction was reduced afterward, while normalized expression distributions became more comparable across datasets (Figures 2A,B; Supplementary Figures S1A, B). In the merged training cohort, we identified 608 DEGs, of which 415 were upregulated and 193 were downregulated. A volcano plot shows their overall distribution (Figure 2C), and a heatmap shows the expression patterns of the 20 most upregulated and 20 most downregulated genes (Figure 2D). Following within-category union and deduplication, the apoptosis, necroptosis/regulated-necrosis, and pyroptosis collections contained 714, 93, and 57 unique genes, respectively. Because these categories overlapped, their cross-category union comprised 781 unique genes. (Figure 2E; Supplementary Table S2); their union comprised 781 unique PRGs. Forty-seven DEGs, including 40 upregulated and 7 downregulated genes, overlapped with this union and were designated DE-PRGs (Figure 2F; Supplementary Figure S1C).

### 3.2 Functional enrichment analysis

**FIGURE 3:**
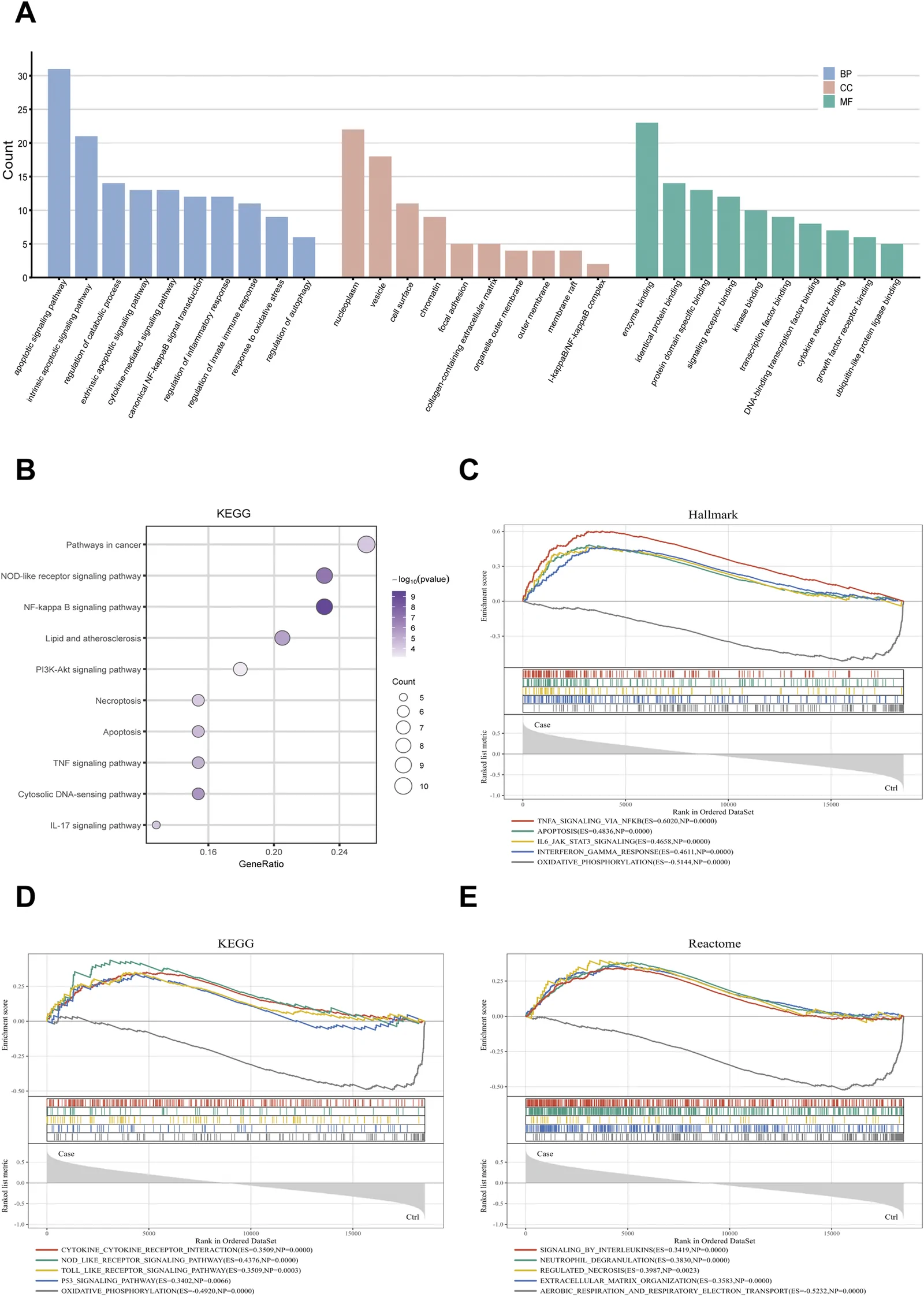
Functional enrichment of PANoptosis-associated differentially expressed genes. The 47 overlapping genes were derived from the training cohort (n = 103; control samples, n = 66; sarcopenia samples, n = 37) **(A)** Gene Ontology enrichment results. Blue, salmon, and teal bars denote biological process, cellular component, and molecular function terms, respectively; bar height indicates gene count **(B)** Kyoto Encyclopedia of Genes and Genomes (KEGG) pathway enrichment. Dot size represents gene count, and color intensity represents −log10(P value) **(C–E)** Gene set enrichment analysis showing representative Hallmark **(C)**, KEGG **(D)**, and Reactome **(E)** gene sets. Colored curves correspond to the pathways listed below each panel. Positive enrichment scores indicate relative enrichment in sarcopenia samples, whereas negative enrichment scores indicate relative enrichment in controls. ES, enrichment score; NP, nominal P value.

We performed GO, KEGG, and GSEA enrichment analyses to characterize the 47 DE-PRGs. GO analysis associated these genes predominantly with apoptosis-related biological processes, including apoptotic signaling and its intrinsic and extrinsic pathways. Cytokine-mediated signaling, canonical NF-κB signaling, regulation of inflammatory responses, innate immune responses, oxidative stress, and autophagy regulation were also represented. At the cellular-component and molecular-function levels, the DE-PRGs were associated mainly with membrane signaling structures, extracellular-matrix components, the inhibitor of NF-κB (IκB)/NF-κB complex, receptor and kinase binding, transcription-factor binding, and ubiquitin-like protein-ligase binding (Figure 3A).

KEGG analysis further associated these genes with interleukin-17 (IL-17), TNF, NF-κB, NOD-like receptor, apoptosis, necroptosis, and phosphoinositide 3-kinase/protein kinase B (PI3K/Akt) signaling pathways, suggesting that inflammatory signaling, programmed cell death, and stress adaptation may intersect in sarcopenia-associated transcriptional changes (Figure 3B). GSEA supported this pattern: sarcopenia samples showed positive enrichment of cytokine–cytokine receptor interaction, NOD-like and Toll-like receptor signaling, TNF-α signaling through NF-κB, interleukin-6/Janus kinase/signal transducer and activator of transcription 3 (IL-6/JAK/STAT3) signaling, interferon-γ response, interleukin signaling, neutrophil degranulation, and regulated necrosis. In contrast, oxidative-phosphorylation and aerobic-respiration pathways were negatively enriched (Figures 3C–E). Thus, the sarcopenia-associated DE-PRG signature was associated with enrichment of inflammatory, programmed-cell-death, extracellular-matrix, and mitochondrial-respiration pathway terms.

### 3.3 Machine-learning identification of consensus PANoptosis-associated candidate genes

**FIGURE 4:**
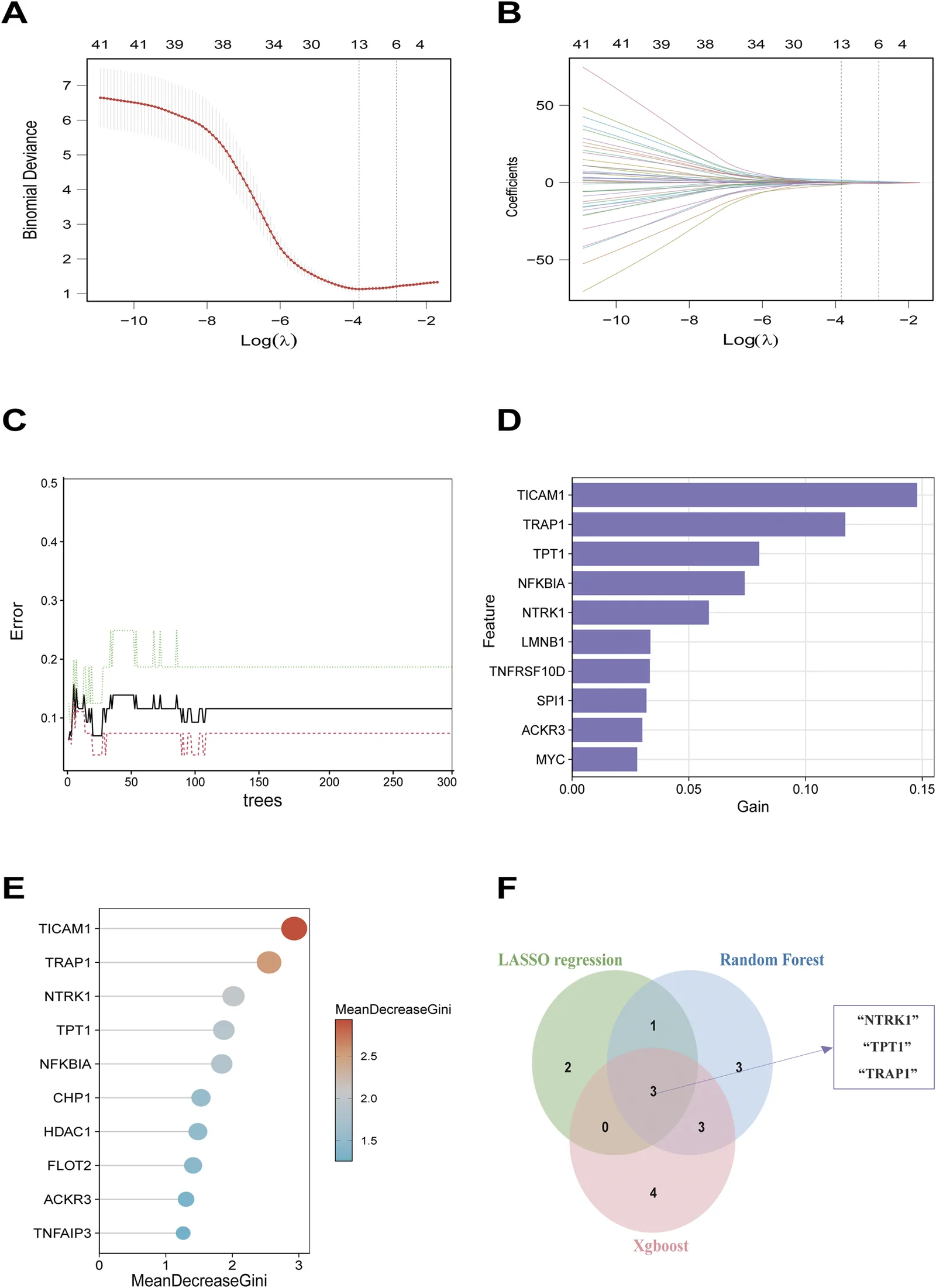
Machine-learning prioritization of PANoptosis-associated candidate genes. Feature selection was performed exclusively in the training cohort (n = 103; control samples, n = 66; sarcopenia samples, n = 37) **(A)** Ten-fold stratified cross-validation curve for least absolute shrinkage and selection operator (LASSO) logistic regression. Red points show mean binomial deviance, gray bars show cross-validation uncertainty, and the values above the plot indicate the number of nonzero coefficients; lambda.1se was used for feature selection **(B)** LASSO coefficient paths across penalty values **(C)** Random-forest classification error across 300 trees **(D)** Top 10 extreme gradient boosting (XGBoost) features ranked by gain; bar length represents feature importance **(E)** Top 10 random-forest features ranked by mean decrease in Gini impurity; horizontal position and color encode importance **(F)** Intersection of the LASSO, random-forest, and XGBoost candidate sets, identifying NTRK1, TPT1, and TRAP1 as consensus candidates.

Within the training cohort, LASSO regression with the lambda.1se criterion retained TRAP1, immediate early response 3 (IER3), TPT1, Sp1 transcription factor (SP1), NTRK1, and calcineurin like EF-hand protein 1 (CHP1) (Figures 4A,B). The RF error curve stabilized as the number of trees increased, and mean decrease in Gini impurity ranked the 10 most important variables (Figures 4C,E). After cross-validation selected the boosting iteration, XGBoost ranked its 10 leading features by gain (Figure 4D). The intersection of the three feature sets yielded TRAP1, TPT1, and NTRK1 as consensus candidates (Figure 4F).

### 3.4 Exploratory discrimination analysis prioritizes TPT1 as a PANoptosis-related candidate marker

**FIGURE 5:**
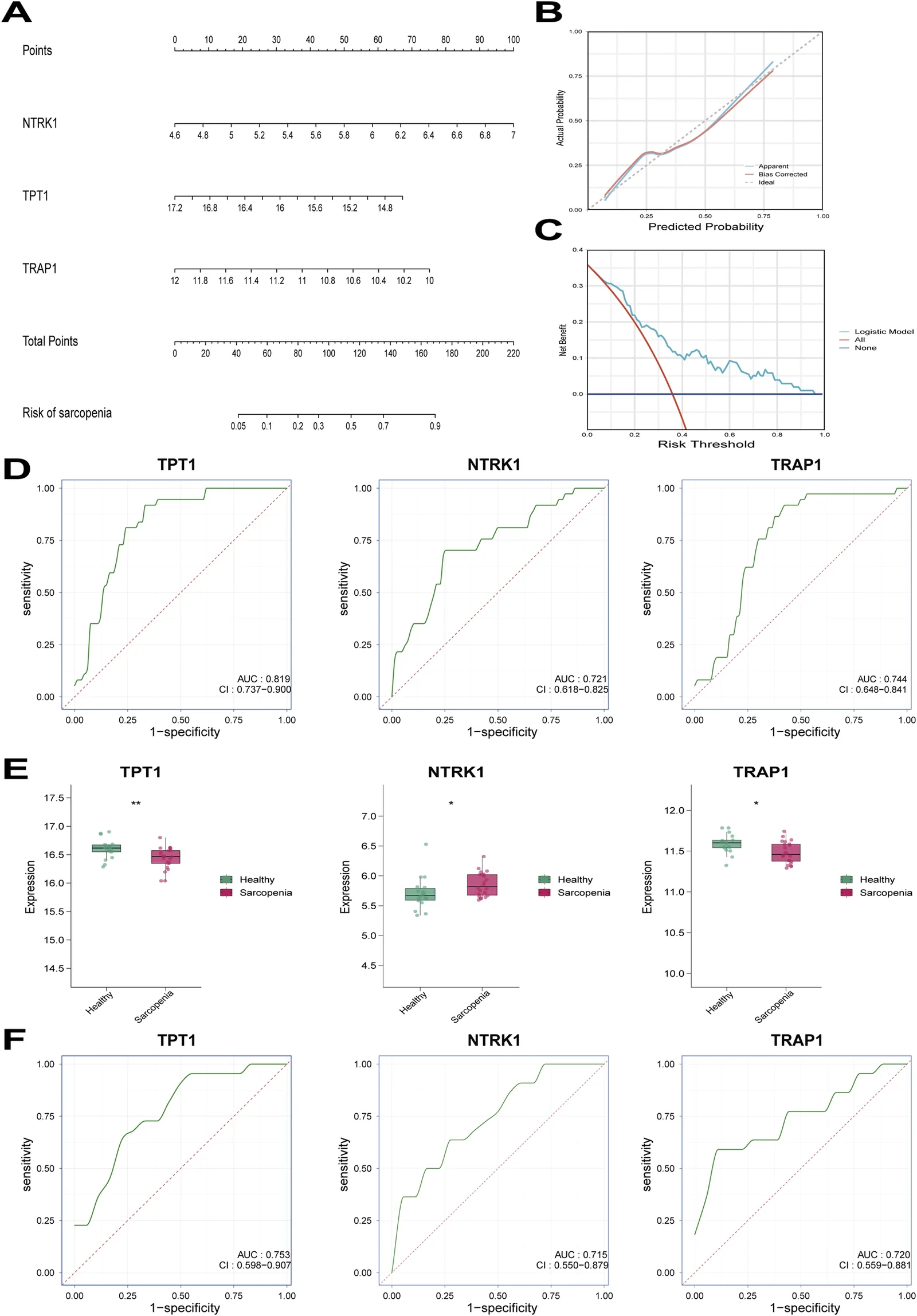
Exploratory evaluation of the three-gene model and individual candidate-gene discrimination **(A)** Nomogram incorporating NTRK1, TPT1, and TRAP1 in the training cohort (n = 103; control samples, n = 66; sarcopenia samples, n = 37) **(B)** Training-cohort calibration plot; blue and red curves show apparent and bias-corrected calibration, respectively, and the gray dashed line represents ideal calibration **(C)** Decision-curve analysis; cyan, red, and blue lines denote the three-gene model, treat-all strategy, and treat-none strategy, respectively **(D)** Receiver operating characteristic (ROC) curves in the training cohort. Areas under the ROC curve (AUCs; 95% DeLong confidence intervals) were 0.819 (0.737–0.900) for TPT1, 0.721 (0.618–0.825) for NTRK1, and 0.744 (0.648–0.841) for TRAP1 **(E)** Candidate-gene expression in the external validation cohort (control samples, n = 20; sarcopenia samples, n = 20). Green and magenta denote control and sarcopenia samples, respectively; points represent individual samples. Boxes show medians and interquartile ranges, and groups were compared with the Wilcoxon rank-sum test **(F)** ROC curves in the external cohort. AUCs (95% DeLong confidence intervals) were 0.753 (0.598–0.907) for TPT1, 0.715 (0.550–0.879) for NTRK1, and 0.720 (0.559–0.881) for TRAP1. *P < 0.05; **P < 0.01.

We next evaluated the sample-level discrimination of NTRK1, TPT1, and TRAP1. A nomogram incorporating all three genes estimated sample-level group-membership probability (Figure 5A). Its calibration curve showed agreement between predicted probabilities and observed outcomes in the training data (Figure 5B). DCA indicated potential net benefit across a limited range of threshold probabilities (Figure 5C).

ROC analysis showed that TPT1 provided the strongest single-gene discrimination in the training cohort, with an AUC of 0.819 (95% CI, 0.737–0.900). NTRK1 and TRAP1 had AUCs of 0.721 and 0.744, respectively (Figure 5D). In the external cohort, TPT1 and TRAP1 were expressed at lower levels in sarcopenia samples, whereas NTRK1 showed higher expression. TPT1 yielded an AUC of 0.753 (95% CI, 0.598–0.907), compared with 0.715 for NTRK1 and 0.720 for TRAP1 (Figures 5E,F). Because TPT1 showed the most consistent discrimination across the two cohorts, we selected it for experimental expression assessment.

### 3.5 Exploratory immune-cell deconvolution

**FIGURE 6:**
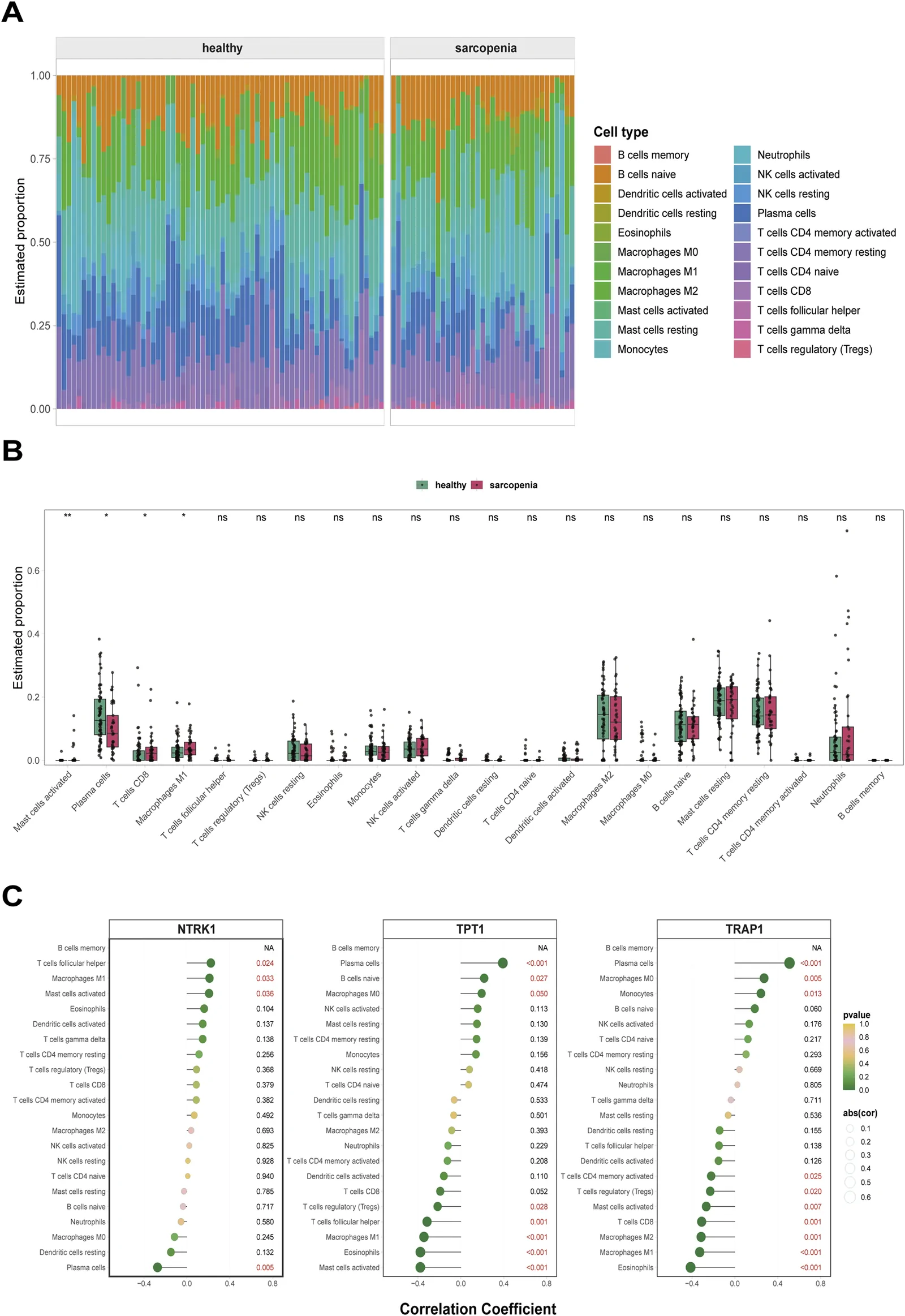
Exploratory CIBERSORT-estimated immune-cell fractions and their associations with candidate-gene expression. Analyses used the training cohort (n = 103; control samples, n = 66; sarcopenia samples, n = 37) **(A)** Relative fractions of 22 LM22 immune-cell types. Each stacked column represents one sample, and colors distinguish estimated cell types **(B)** Comparison of estimated fractions between control and sarcopenia samples. Green and magenta denote the two groups; points represent individual samples, and groups were compared with the Wilcoxon rank-sum test **(C)** Spearman correlations between NTRK1, TPT1, or TRAP1 expression and estimated immune-cell fractions. Horizontal position represents the correlation coefficient, dot size represents its absolute value, and dot color represents the nominal P value. NA (not available) indicates that a correlation could not be estimated because of insufficient variability. Ns, not significant; *P < 0.05; **P < 0.01.

We used CIBERSORT to estimate the relative proportions of 22 immune-cell types in the training cohort. The stacked bar plot showed substantial intersample heterogeneity in estimated immune-cell composition in both control and sarcopenia samples (Figure 6A). Exploratory group comparisons suggested potential differences in activated mast cells, plasma cells, cluster of differentiation 8-positive (CD8^+^) T cells, and M1 macrophages, whereas most other subsets showed no clear trend (Figure 6B). Plasma-cell estimates tended to be lower in sarcopenia samples, while CD8^+^ T-cell and M1-macrophage estimates tended to be higher; these findings indicate differences in selected model-estimated fractions rather than global immune-cell changes. Correlation analysis further identified potential associations between candidate-gene expression and specific estimated subsets. NTRK1 correlated positively with T follicular helper cells, M1 macrophages, and activated mast cells and negatively with plasma cells. TPT1 correlated positively with plasma cells and naïve B cells and negatively with activated mast cells, eosinophils, M1 macrophages, T follicular helper cells, and regulatory T cells (Tregs). TRAP1 correlated positively with plasma cells, M0 macrophages, and monocytes and negatively with eosinophils, M1 and M2 macrophages, CD8^+^ T cells, activated mast cells, Tregs, and activated cluster of differentiation 4-positive (CD4^+^) memory T cells (Figure 6C). These computational estimates are hypothesis-generating and require validation in independent cohorts with orthogonal methods such as multiplex immunohistochemistry or flow cytometry.

### 3.6 Single-nucleus transcriptomic landscape of aging skeletal muscle and candidate-gene expression

**FIGURE 7:**
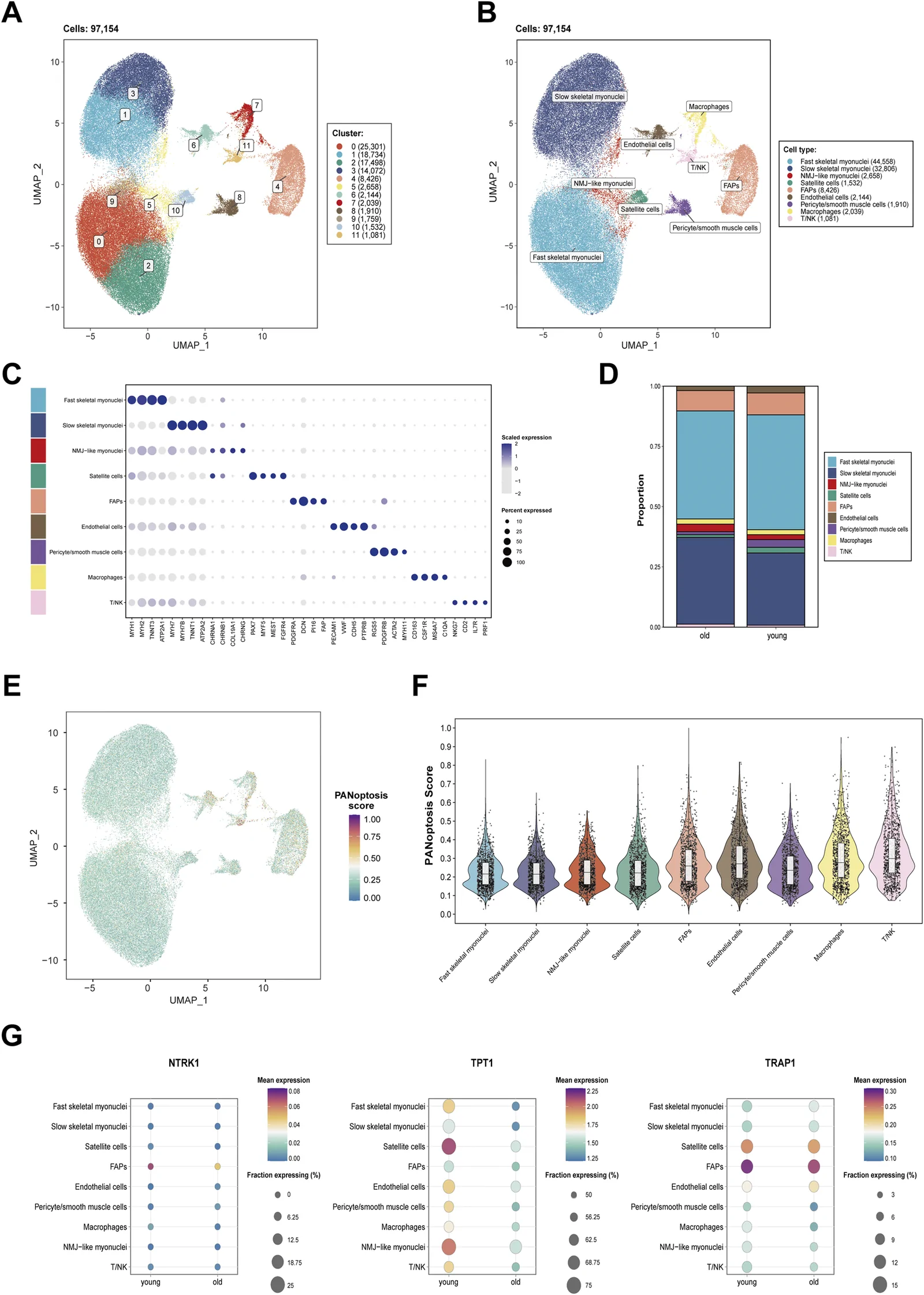
Aging-related single-nucleus context of the PANoptosis-associated module score and candidate-gene expression in human skeletal muscle. GSE167186 included 17 donors (young, n = 6; older, n = 11) and 97,154 nuclei retained after quality control **(A)** Uniform Manifold Approximation and Projection (UMAP) visualization of 12 transcriptional clusters **(B)** UMAP visualization after merging clusters into nine annotated cell classes. Colors identify cell classes, and the legend reports corresponding numbers of nuclei **(C)** Canonical marker-gene dot plot. Dot size indicates the percentage of nuclei expressing each marker, and color indicates scaled expression **(D)** Descriptive pooled cell-type composition in the older and young groups **(E)** UMAP distribution of the 50-gene PANoptosis-related module score; color represents the score **(F)** Module-score distributions across the 9 cell classes **(G)** NTRK1, TPT1, and TRAP1 expression by cell class and age group. Dot size indicates the fraction of expressing nuclei, and color indicates mean expression. FAPs, fibro-adipogenic progenitors; NMJ, neuromuscular junction; T/NK, T and natural killer cells.

We reanalyzed GSE167186, which compares skeletal muscle from young and older individuals rather than clinically defined sarcopenia and control groups. After quality control and doublet removal, 97,154 nuclei from 17 donors remained for analysis (Supplementary Figures S2A, B). Graph-based clustering partitioned the nuclei into 12 transcriptional clusters, visualized by UMAP (Figure 7A). Using established skeletal-muscle markers (Figures 7B,C; Supplementary Figure S2C), we annotated fast skeletal myonuclei (MYH1, MYH2, TNNT3, ATP2A1), slow skeletal myonuclei (MYH7, MYH7B, TNNT1, ATP2A2), neuromuscular-junction (NMJ)-like myonuclei (CHRNA1, CHRNB1, COL19A1, CHRNG), satellite cells (PAX7, MYF5, MEST, FGFR4), fibro-adipogenic progenitors (FAPs; PDGFRA, DCN, PI16, FAP), endothelial cells (PECAM1, VWF, CDH5, PTPRB), pericyte/smooth-muscle cells (RGS5, PDGFRB, ACTA2, MYH11), macrophages (CD163, CSF1R, MS4A7, C1QA), and T-cell/natural killer (T/NK)-cell populations (NKG7, CD2, IL7R, PRF1). We then merged clusters with similar identities into nine major populations (Figure 7B) and quantified their relative proportions in young and older muscle (Figure 7D; Supplementary Figure S2D). Fast and slow skeletal myonuclei dominated both age groups, whereas stromal and immune populations formed smaller, distinct compartments. Older muscle showed descriptive shifts toward greater representation of slow myonuclei and immune-related compartments and lower representation of fast myonuclei, consistent with age-associated multicellular remodeling.

We next calculated an aggregate PANoptosis-related module score at single-nucleus resolution. Scores were broadly distributed across the UMAP space and tended to be higher in macrophages, T/NK cells, endothelial cells, and FAPs than in the major fast and slow myonuclear populations (Figures 7E,F). These expression-based scores suggest that the selected PANoptosis-related transcriptional program spans immune, stromal, vascular, and myonuclear compartments; they do not demonstrate execution of PANoptosis in any cell type.

We then examined the single-nucleus expression of NTRK1, TPT1, and TRAP1. Feature and dot plots showed sparse NTRK1 expression, TRAP1 expression in selected populations, and broad TPT1 expression across myonuclear, satellite-cell, stromal, endothelial, macrophage, and T/NK-cell compartments (Figure 7G; Supplementary Figure S2E). Compared with young samples, older samples showed lower TPT1 expression intensity and/or a smaller fraction of TPT1-expressing nuclei across several annotated populations. These observations provide aging-related cellular localization and do not establish that the TPT1 pattern is specific to sarcopenia.

### 3.7 Exploratory network, regulatory, compound, and pathway analyses

**FIGURE 8:**
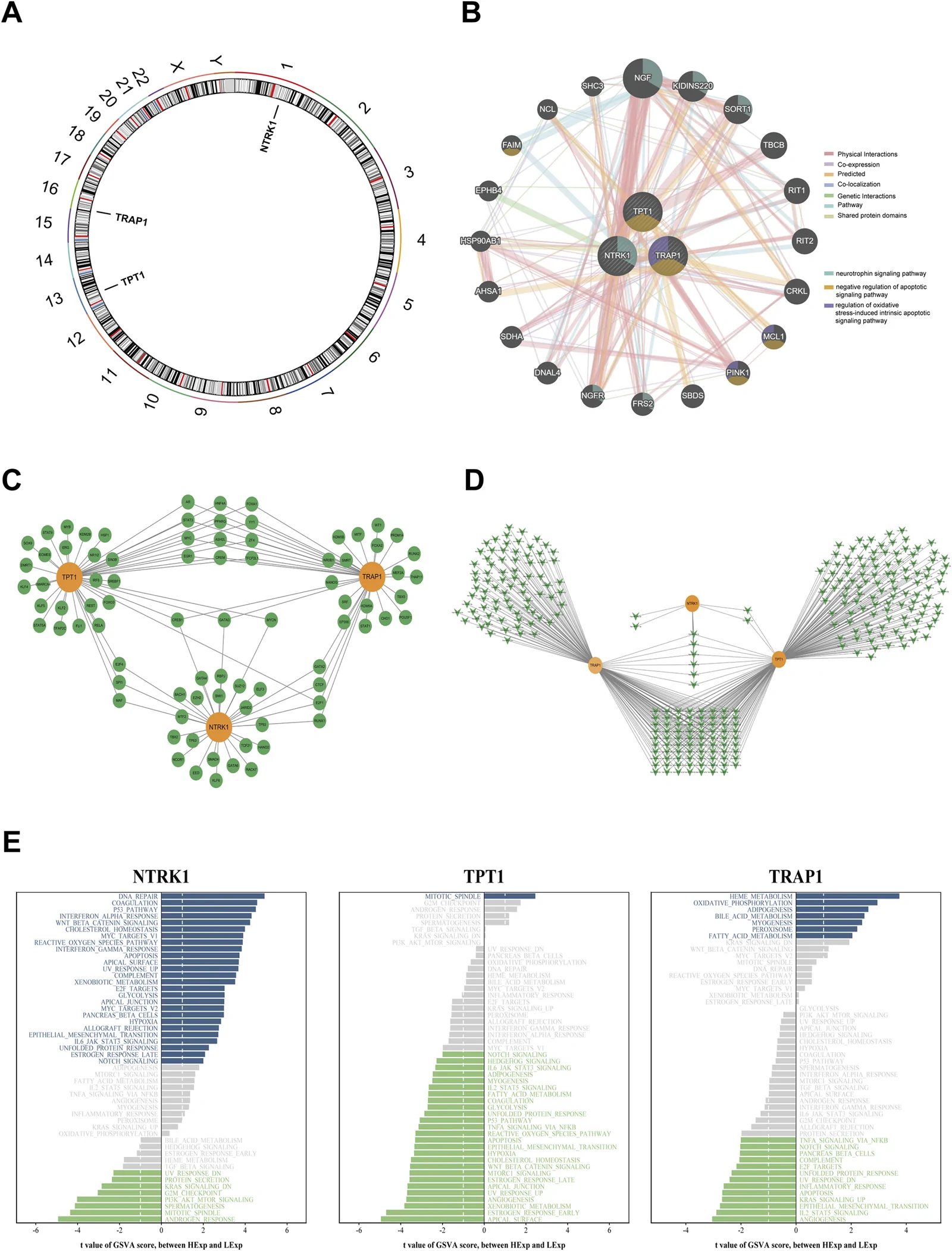
Exploratory network, regulatory, and pathway-score analyses of the candidate genes **(A)** Circular chromosomal ideogram showing the locations of NTRK1, TPT1, and TRAP1 **(B)** GeneMANIA functional-association network containing the three query genes and 20 predicted functional partners. Edge colors distinguish physical interaction, co-expression, predicted interaction, co-localization, genetic interaction, pathway, and shared-domain evidence. Colored node sectors indicate representative pathway memberships, including neurotrophin signaling, negative regulation of apoptotic signaling, and regulation of oxidative-stress-induced intrinsic apoptotic signaling; gray nodes lack these highlighted annotations **(C)** Predicted transcription factor (TF)–gene network. Orange nodes denote candidate genes, green nodes denote TFs, and gray lines denote predicted relationships **(D)** Predicted microRNA (miRNA)–gene network. Orange nodes denote candidate genes, green V-shaped nodes denote miRNAs, and gray lines denote predicted relationships **(E)** Gene set variation analysis (GSVA) in the training cohort (n = 103) after median stratification by candidate-gene expression. Bars show Welch t statistics comparing high- and low-expression groups. Blue positive bars indicate higher scores in the high-expression group, green negative bars indicate higher scores in the low-expression group, and gray bars denote pathways that did not meet the exploratory thresholds of |t| > 1 and nominal P < 0.05.

To characterize the biological context of the three candidate genes, we performed chromosomal mapping, GeneMANIA interaction-network analysis, upstream regulatory-network prediction, DSigDB compound-signature analysis, and single-gene GSVA. TPT1, TRAP1, and NTRK1 mapped to chromosomes 13, 16, and 1, respectively (Figure 8A), indicating that they represent distinct genomic loci rather than a chromosomal gene cluster.

GeneMANIA connected the candidate genes to predicted functional partners, including NGF, KIDINS220, SORT1, MCL1, PINK1, FRS2, NGFR, HSP90AB1, and SDHA (Figure 8B). Network annotations implicated neurotrophin signaling, negative regulation of apoptosis, and oxidative-stress-related intrinsic apoptotic signaling. Predicted TF–gene and miRNA–gene networks contained 82 TFs and 107 edges and 281 miRNAs and 372 edges, respectively (Figures 8C,D; Supplementary Tables S5, S6). CREB1, E2F1, and GATA3 were shared predicted TFs, and six miRNAs were predicted to target all three candidate genes. The DSigDB analysis identified 106 compound or chemical-perturbagen signature terms with adjusted P < 0.05 (Supplementary Table S7); Table 1 lists the five highest-ranked terms.

**TABLE 1:** Top five compound or chemical-perturbagen signatures associated with the candidate-gene set in the Drug Signatures Database (DSigDB).

Term	Adjusted P-value	Combined Score	Associated candidate gene(s)
Nickelous acetate CTD 00003684	0.0035888674896012176	7100.033933987403	TRAP1; TPT1
Dronabinol CTD 00006853	0.02882598120345684	659.5709003271993	TRAP1; TPT1
Potassium dichromate CTD 00006598	0.02882598120345684	427.2712025582741	TRAP1; TPT1
CHEMBL1967116 roche	0.02882598120345684	2877.3716896348615	NTRK1
Gambogic acid CTD 00002247	0.02882598120345684	2718.114679972292	NTRK1

Single-gene-stratified GSVA further characterized pathway associations. Lower TPT1 expression coincided with higher scores for apoptosis, reactive oxygen species (ROS), TNF-α/NF-κB, IL-6/JAK/STAT3, p53, unfolded-protein-response, mechanistic target of rapamycin complex 1 (mTORC1), hypoxia, and glycolysis pathways (Figure 8E). TRAP1 showed weaker associations: oxidative phosphorylation was higher in the TRAP1-high group, whereas apoptosis- and inflammation-related pathways were modestly higher in the TRAP1-low group. Higher NTRK1 expression was tentatively associated with DNA repair, p53, interferon response, ROS, and apoptosis pathways. Thus, the candidate genes, particularly TPT1, were associated with inflammatory, oxidative-stress, and cell-death-related expression patterns.

### 3.8 TPT1 is downregulated in a D-galactose-induced muscle-wasting model

**FIGURE 9:**
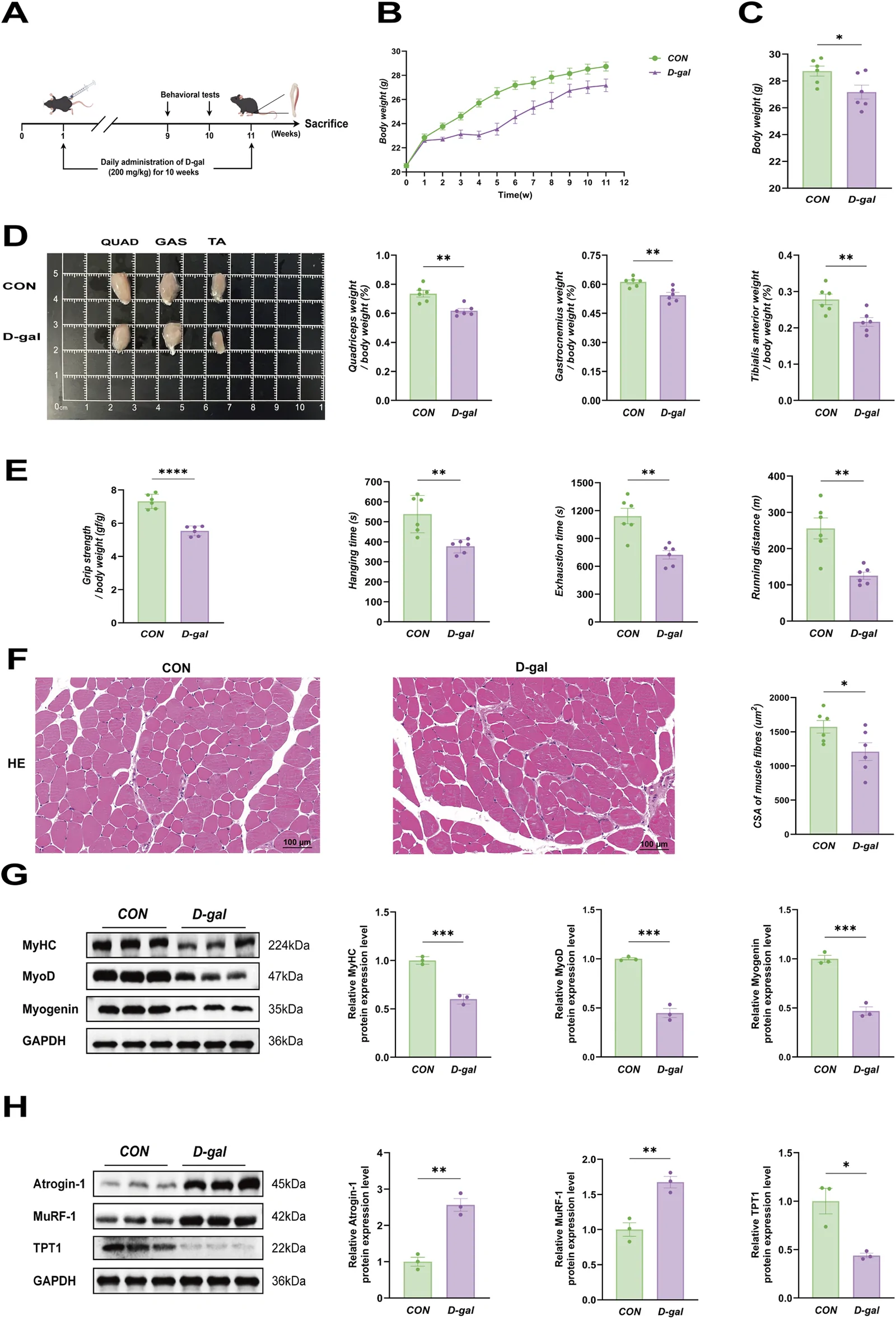
Reduced TPT1 accompanies D-galactose-induced muscle-wasting phenotypes *in vivo* **(A)** Experimental timeline showing daily D-galactose administration (200 mg/kg) for 10 weeks, behavioral testing, and tissue collection **(B)** Weekly body-weight changes **(C)** Final body weight **(D)** Representative gross images of quadriceps (QUAD), gastrocnemius (GAS), and tibialis anterior (TA) muscles and their weights normalized to body weight **(E)** Grip strength normalized to body weight, hanging time, exhaustion time, and running distance **(F)** Representative hematoxylin-and-eosin (H&E)-stained gastrocnemius sections and quantification of myofiber cross-sectional area (CSA); scale bars, 100 μm. For B–F, n = 6 mice per group **(G)** Representative western blots and densitometric quantification of myosin heavy chain (MyHC), myogenic differentiation 1 (MyoD), and myogenin **(H)** Representative western blots and densitometric quantification of Atrogin-1, muscle RING-finger protein 1 (MuRF-1), and TPT1. Glyceraldehyde-3-phosphate dehydrogenase (GAPDH) served as the loading control. For G and H, n = 3 biologically independent muscle samples per group. Green denotes vehicle controls and purple denotes the D-galactose group; each dot represents one independent replicate. Data are presented as mean ± SD. *P < 0.05; **P < 0.01; ***P < 0.001; ****P < 0.0001.

To validate the bioinformatics findings, we used D-galactose treatment to model oxidative stress, senescence-like changes, and muscle wasting *in vivo*. Compared with control mice, D-galactose-treated mice showed reduced body weight gain and lower final body weight, indicating an aging-related wasting phenotype (Figures 9A–C). Consistently, the relative weights of major hindlimb muscles, including the quadriceps, gastrocnemius, and tibialis anterior, were significantly decreased after D-galactose administration (Figure 9D). Functional assessments further revealed impaired skeletal muscle performance, as evidenced by reduced grip strength, shorter hanging time, decreased exhaustion time, and reduced running distance (Figure 9E). Histological examination of gastrocnemius muscle showed a marked reduction in myofiber cross-sectional area in D-galactose-treated mice, supporting the presence of skeletal muscle atrophy (Figure 9F).

We next examined whether these phenotypic changes were accompanied by alterations in muscle-related molecular markers. Western blot analysis showed that the expression levels of MyHC, MyoD, and Myogenin were reduced in gastrocnemius muscle from D-galactose-treated mice, whereas the atrophy-related proteins Atrogin-1 and MuRF-1 were increased (Figures 9G,H). Importantly, TPT1 protein expression was significantly decreased in the D-galactose group compared with the control group (Figure 9H). These results indicate that TPT1 downregulation is associated with D-galactose-induced skeletal muscle atrophy and functional decline *in vivo*.

### 3.9 D-galactose-induced senescent C2C12 cells exhibit reduced TPT1 expression

**FIGURE 10:**
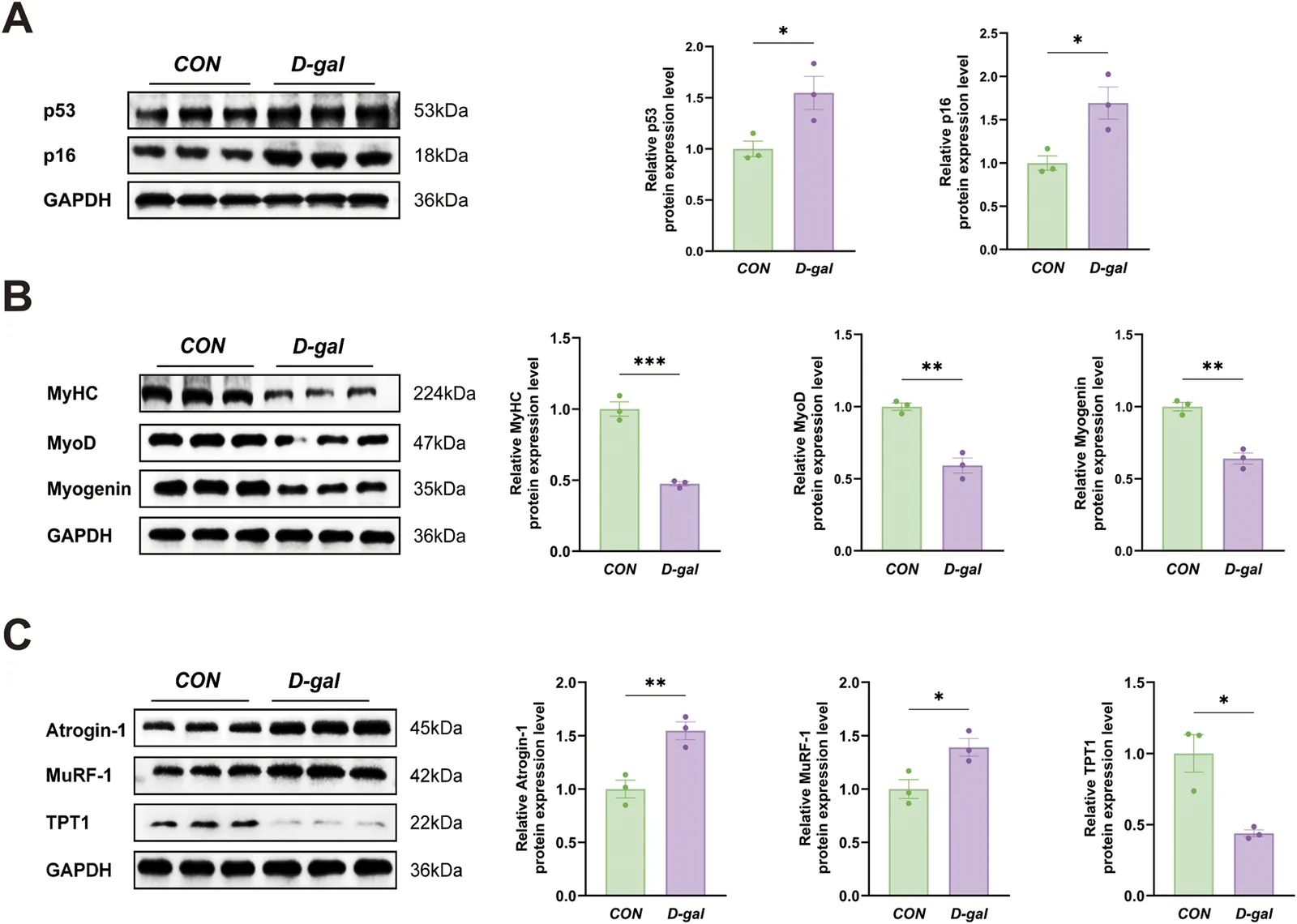
D-galactose-treated C2C12 myotubes show senescence- and atrophy-associated protein changes accompanied by reduced TPT1 **(A)** Representative western blots and densitometric quantification of p53 and p16 **(B)** Representative western blots and densitometric quantification of myosin heavy chain (MyHC), myogenic differentiation 1 (MyoD), and myogenin **(C)** Representative western blots and densitometric quantification of Atrogin-1, muscle RING-finger protein 1 (MuRF-1), and TPT1. Glyceraldehyde-3-phosphate dehydrogenase (GAPDH) served as the loading control. For A–C, n = 3 independent experiments per group. Green denotes controls and purple denotes the D-galactose group; each dot represents one independent experiment. Data are presented as mean ± SD. *P < 0.05; **P < 0.01; ***P < 0.001.

To determine whether the reduction in TPT1 expression could also be observed *in vitro*, C2C12 cells were treated with D-galactose to establish a cellular model of sarcopenia-related senescence. D-galactose exposure increased the protein levels of the senescence-associated markers p53 and p16, indicating the induction of a senescence-like phenotype (Figure 10A). In parallel, the expression levels of MyHC, MyoD, and Myogenin were decreased, whereas Atrogin-1 and MuRF-1 were increased, suggesting impaired myogenic features and activation of muscle atrophy-related molecular changes (Figures 10B,C). Consistent with the bulk transcriptomic results and the *in vivo* findings, TPT1 protein expression was markedly reduced in D-galactose-treated C2C12 cells (Figure 10C). Together, these results demonstrate that TPT1 downregulation is consistently observed in both D-galactose-induced cellular and animal models of sarcopenia-related muscle wasting.

## 4 Discussion

Sarcopenia is a systemic age-related disorder characterized by losses of muscle mass, strength, and physical performance, with major consequences for frailty, disability, and mortality (Sayer et al., 2024; Larsson et al., 2019). Multi-omic and single-cell studies have extended the field beyond a myofiber-centered view by showing coordinated remodeling of metabolic programs, muscle stem cells, FAPs, vascular cells, immune cells, and specialized myonuclear states (Kedlian et al., 2024; Lai et al., 2024). Consistent with this broader framework, our DE-PRG enrichment results encompassed cytokine signaling, NF-κB and NOD-like receptor activation, TNF signaling, necroptosis, extracellular-matrix remodeling, autophagy, and impaired oxidative phosphorylation. The signature therefore represents a composite inflammatory-stress transcriptional program rather than direct evidence for activation of a single death-execution mechanism (Moiseeva et al., 2023; Walter et al., 2024).

Prior evidence linking individual PANoptosis components to muscle atrophy and aging supports this interpretation. The NLR family pyrin domain containing 3 (NLRP3) inflammasome contributes to age-related muscle loss in mice, and experiments in C2C12 myotubes have linked NLRP3 activity to morphological and metabolic changes, mitochondrial association, and ROS production (McBride et al., 2017; Eggelbusch et al., 2022; Swanson et al., 2019). TNF-α can promote sarcopenia-related changes through caspase-8/caspase-3/gasdermin E (GSDME)-mediated pyroptosis, illustrating functional overlap between apoptotic caspases and gasdermin-dependent inflammatory death in muscle (Wu et al., 2023). Autophagy dysregulation, ferroptosis, and impaired mitochondrial quality control also contribute to muscle aging (Han et al., 2022; Xie et al., 2023; Ru et al., 2025; Affourtit and Carré, 2024). Together with our enrichment results, these observations support convergent remodeling of inflammatory cell-death and stress-adaptive pathways in sarcopenic muscle.

Each of the three machine-learning derived candidate- NTRK1, TRAP1, and TPT1, has biological plausibility in sarcopenia. NTRK1 encodes tropomyosin receptor kinase A (TrkA), which participates in neurotrophin signaling, neuronal survival, and differentiation. Because neuromuscular-junction instability, altered motor innervation, and impaired neurotrophic support characterize age-related muscle decline, lower NTRK1 expression may reflect disruption of the neuromuscular axis (Li et al., 2018). TRAP1 encodes a mitochondrial heat shock protein 90 (Hsp90)-family chaperone that regulates mitochondrial proteostasis, oxidative-stress responses, metabolism, and cell-death susceptibility (Cannino et al., 2022). Because skeletal muscle depends heavily on mitochondrial energy metabolism, lower TRAP1 expression may indicate reduced mitochondrial resilience under stress.

TPT1, also known as translationally controlled tumor protein (TCTP), is a conserved regulator of cell growth, survival, stress adaptation, apoptosis, autophagy, and immune signaling (Bommer and Telerman, 2020). Intracellular TPT1 can oppose Bcl-2-associated X protein (BAX)-dependent mitochondrial apoptosis (Susini et al., 2008). Studies in non-muscle systems also indicate that TPT1 can regulate autophagy through the Beclin 1 (BECN1) interactome and mTORC1/AMP-activated protein kinase (AMPK) signaling, although the direction and consequences depend on cellular context (Bae et al., 2017). Intracellular TPT1 generally supports survival and adaptation, whereas extracellular TPT1 can act as a histamine-releasing and pro-inflammatory factor. In our data, lower TPT1 expression coincided with higher GSVA scores for apoptosis, ROS, TNF-α/NF-κB, IL-6/JAK/STAT3, p53, unfolded-protein response, hypoxia, glycolysis, and mTORC1 pathways. This coordinated pattern is compatible with altered cellular stress tolerance, but it cannot determine whether TPT1 loss precedes, follows, or compensates for muscle injury.

The GeneMANIA, TF/miRNA, GSVA, immune-deconvolution, and DSigDB analyses provide complementary context for this association. GeneMANIA connected the candidate genes to proteins involved in neurotrophin signaling, mitochondrial stress responses, and apoptosis, while the predicted regulatory networks identified candidate upstream factors shared by TPT1, TRAP1, and NTRK1. CIBERSORT suggested differences in selected model-estimated immune-cell fractions and correlations between TPT1 expression and several estimated subsets. These database- and expression-derived relationships can guide targeted experiments but do not establish direct regulation, cell-to-cell communication, target engagement, or therapeutic efficacy. Likewise, compound-signature associations generate hypotheses and should not be interpreted as clinically actionable treatments.

The single-nucleus analysis localized TPT1 across myonuclear, satellite-cell, stromal, endothelial, and immune compartments and showed lower expression in older skeletal muscle. This multicellular distribution is relevant because age-related muscle dysfunction reflects impaired coordination among contractile, regenerative, vascular, stromal, and immune compartments. However, GSE167186 compares young and older donors and does not include a clinically phenotyped sarcopenia group. These results therefore provide aging-related cellular context rather than sarcopenia-specific evidence.

The D-galactose experiments reproduced lower TPT1 protein abundance alongside muscle-wasting and senescence-like changes. D-galactose can model oxidative stress and selected aging-like phenotypes in skeletal muscle, but it does not reproduce the gradual endocrine, neuromuscular, metabolic, and immune changes of natural aging or the clinical diagnostic construct of sarcopenia (Yanar et al., 2019). Moreover, co-occurring expression changes cannot establish whether lower TPT1 is causal, downstream, or compensatory.

Several limitations should be acknowledged. First, the clinical analyses relied mainly on retrospective public datasets; prospective cohorts with harmonized diagnostic criteria, balanced covariates, and prespecified validation procedures are required before the clinical utility of TPT1 can be assessed. Second, the D-galactose model represents oxidative-stress-induced, aging-like muscle wasting rather than natural aging or clinically defined sarcopenia, and the animal experiment included only male mice. Future studies should use naturally aged animals and include both sexes. Third, the single-nucleus dataset characterizes skeletal-muscle aging rather than phenotyped sarcopenia, so its findings cannot be considered disease specific. Fourth, the experiments did not measure canonical execution markers of apoptosis, pyroptosis, and necroptosis or perturb TPT1 expression. Future work should combine TPT1 knockdown and overexpression with myotube-size, viability, and cell-death assays; cleaved-caspase and gasdermin measurements; NLRP3 and apoptosis-associated speck-like protein containing a caspase-recruitment domain (ASC) readouts; receptor-interacting protein kinases 1 and 3 (RIPK1 and RIPK3) and phosphorylated mixed lineage kinase domain-like protein (p-MLKL) measurements; and cell-type-resolved analyses. These experiments are needed to determine whether TPT1 directly modifies muscle wasting or coordinated inflammatory cell death.

## 5 Conclusion

In conclusion, this study identifies a PANoptosis-associated transcriptional signature in sarcopenia and prioritizes TPT1 as a candidate marker associated with the bulk-transcriptomic sarcopenia phenotype. Lower TPT1 transcript expression was observed in the integrated and external bulk-transcriptomic cohorts and in human single-nucleus aging data, while lower TPT1 protein abundance accompanied D-galactose-induced changes in mouse and C2C12 models. The aging-muscle, regulatory-network, pathway, compound-signature, and induced-model analyses provide exploratory context and testable hypotheses, but prospective clinical validation and targeted functional studies are required to establish disease specificity, clinical utility, and biological causality.

## Data Availability

The original contributions presented in the study are included in the article/supplementary material, further inquiries can be directed to the corresponding authors.
