# Supplementary material for: Integrative transcriptomic and experimental analyses prioritize TPT1 as a PANoptosis-associated candidate molecular marker in sarcopenia
Source: Front Cell Dev Biol. 2026 Aug 20;14:1912393. doi: 10.3389/fcell.2026.1912393 (PMC13490725; doi:10.3389/fcell.2026.1912393)
Supplement: Supplementary file 1 [file DataSheet1.zip › Supplementary Figures S1–S3.docx]

**Supplementary Figure 1** (A, B) Boxplot distributions of gene expression values across four GEO datasets before (A) and after (B) batch effect correction.(C) Expression of the 47 overlapping genes.

**Supplementary Figure 2** (A, B) Violin plots showing per-sample quality-control metrics, including nCount_RNA, nFeature_RNA, and mitochondrial transcript percentage, before and after quality-control filtering. (C) Dot plot of canonical marker genes across the 12 transcriptional clusters, supporting downstream cell-type annotation. Dot size indicates the percentage of nuclei expressing each marker, and color indicates scaled expression. (D) Sample-level cell-type composition across individual young and old donors. (E) UMAP FeaturePlot visualization of NTRK1, TPT1, and TRAP1 expression. percent.mt, mitochondrial transcript percentage.

**Supplementary Figure 3** **(A)** Gross anatomical image of the dissected mouse lower limbs, showing the overall morphology of hindlimb skeletal muscles.**(B)** Representative image of the treadmill running test used to evaluate exercise endurance and locomotor performance in mice.**(C)** Representative image of the inverted grid hanging test used to assess limb grip strength and balance ability.
