# Supplementary material for: Integrative transcriptomic and experimental analyses prioritize TPT1 as a PANoptosis-associated candidate molecular marker in sarcopenia
Source: Front Cell Dev Biol. 2026 Aug 20;14:1912393. doi: 10.3389/fcell.2026.1912393 (PMC13490725; doi:10.3389/fcell.2026.1912393)
Supplement: Supplementary file 1 [file DataSheet1.zip › Supplementary_Table_S1.docx]

**Table S1. Dataset details used in this study**

| **GEO accession** | **Original study/cohort; age and sex** | **Organism, sample source and biopsy site** | **Platform / experiment type** | **Original GEO design, diagnostic criteria and potential confounders** | **Samples used in current analysis** | **Time** |
| --- | --- | --- | --- | --- | --- | --- |
| **GSE111006** *Training cohort* | Hertfordshire Sarcopenia Study (HSS), UK; Caucasian community-dwelling older men; subseries of GSE111017/MEMOSA. All male. Age: controls 72.6 ± 2.3 years (68.4–76.7); sarcopenia 74.3 ± 3.6 years (69.0–76.8).  Migliavacca et al., Nature Communications 2019 (PMID: 31862890). | Homo sapiens; vastus lateralis muscle biopsy. Semi-open biopsy using a Weil–Blakesley conchotome after an overnight fast; tissue was snap-frozen and stored at −80 °C. | GPL16791; Illumina HiSeq 2500; total RNA-seq; TruSeq Stranded RNA Ribo-Zero Gold. HSS RNA was isolated using the mirVana miRNA Isolation Kit. | 40 GEO samples: 28 healthy controls, 4 sarcopenia, 5 low-muscle-mass only, and 3 low-strength and/or low-performance only. EWGSOP (2010): appendicular lean mass index (ALMI) ≤7.23 kg/m² plus grip strength <30 kg and/or gait speed ≤0.8 m/s; muscle mass measured by DXA and gait by a 3-m walk.  **Potential confounders:** only 4 sarcopenia cases, male-only Caucasian cohort, and cohort-specific RNA extraction and functional testing. Active ischemic heart disease, diabetes, myopathy, and neuromuscular conditions affecting the legs were excluded in the source cohort. | 4 sarcopenia vs 28 healthy controls. Participants with isolated low muscle mass or isolated low strength/performance were excluded. | Public 2019 |
| **GSE111010** *Training cohort* | Jamaica Sarcopenia Study (JSS); Afro-Caribbean community-dwelling older men; subseries of GSE111017/MEMOSA. All male. Age: controls 76.4 ± 7.4 years (64–89); sarcopenia 76.8 ± 7.9 years (65–89).  Migliavacca et al., Nature Communications 2019 (PMID: 31862890). | Homo sapiens; mid-vastus lateralis muscle biopsy, approximately 12 cm above the patella, using a 5-mm Bergström needle after a 12-h fast and 24–36 h after structured exercise; tissue was frozen in cooled isopentane and stored at −80 °C. | GPL16791; Illumina HiSeq 2500; total RNA-seq; TruSeq Stranded RNA Ribo-Zero Gold. RNA was isolated using QIAzol followed by miRNeasy purification. | 39 GEO samples: 14 healthy controls, 9 sarcopenia, 5 low-muscle-mass only, and 11 low-strength and/or low-performance only. EWGSOP (2010): ALMI ≤7.23 kg/m² plus grip strength <30 kg and/or gait speed ≤0.8 m/s; DXA, Lafayette dynamometer, and gait speed derived from the 6-min walk test. **Potential confounders:** Afro-Caribbean ancestry, broad age range, and DXA/dynamometer/gait protocols different from HSS; detailed comorbidity covariates are not provided in GEO. | 9 sarcopenia vs 14 healthy controls. Participants with isolated low muscle mass or isolated low strength/performance were excluded. | Public 2019 |
| **GSE226151** *Training cohort* | West China Hospital/Sichuan University Chinese cohort; age- and sex-matched by the source study design. Each selected group included 10 women and 10 men. Age: healthy aged 67.7 ± 6.9 years (53–80); sarcopenia 71.6 ± 8.2 years (55–86). Zuo et al., Nature Aging 2025 (PMID: 39910243). | Homo sapiens; vastus lateralis muscle biopsy according to the original article (GEO annotates the source as skeletal muscle). Biopsies were promptly frozen in liquid nitrogen and stored at −80 °C. | GPL16791; Illumina HiSeq 2500; SMARTER mRNA-seq library preparation; RNeasy Mini Plus RNA isolation; processed using CLC Genomics Workbench v11.0.1. | 60 GEO samples: 20 healthy aged, 20 possible sarcopenia, and 20 sarcopenia. AWGS 2019 criteria were applied using BIA-derived skeletal muscle index, grip strength, and gait speed; the source study classified sarcopenia when low muscle mass and low muscle strength coexisted.  **Potential confounders:** hospital-based Chinese cohort and both sexes; BMI was markedly lower in sarcopenia than in healthy aged participants (21.0 ± 2.9 vs 25.7 ± 2.8 kg/m²), with additional differences in albumin and limb anthropometry. | 20 sarcopenia vs 20 healthy aged controls. The 20 possible-sarcopenia samples were excluded. | Public 2024 |
| **GSE238215** *Training cohort* | Skeletal Muscle-On-A-Chip in Microgravity as a Platform for Regeneration Modeling and Drug Screening. The clinical comparison subset comprised banked human vastus lateralis muscle total-RNA samples obtained from the University of Kentucky Center for Muscle Biology under Institutional Review Board approval (Protocol No. 46746).  The sarcopenia group was aged 64–75 years (mean, 70.25 years), whereas the healthy group was aged 48–71 years (mean, 55.75 years).  Kim et al., Stem Cell Reports 2024 (PMID: 39059375). | Homo sapiens; banked total RNA obtained from human vastus lateralis muscle tissue. The clinical subset included four donors classified as having sarcopenia and four healthy donors, with two females and two males in each group. | GPL20301; Illumina HiSeq 4000; RNA libraries prepared by Novogene; AllPrep Mini Kit; alignment to hg38 with HISAT2 v2.0.5 and quantification with featureCounts v1.5.0-p3. The healthy group included three middle-aged donors aged 48, 50, and 54 years, limiting comparability with predominantly older cohorts. | 16 GEO samples: 8 muscle-on-a-chip samples and 8 human vastus lateralis samples (4 labelled sarcopenia and 4 labelled normal). Sarcopenia was defined using sex-specific relative skeletal muscle index thresholds, calculated as muscle mass divided by height squared: RSM <5.46 kg/m² for females and <7.26 kg/m² for males. Muscle strength and physical-performance criteria were not reported or incorporated into the classification.  **Potential confounders:**Sex was balanced between groups，but the sample size was small, and information on muscle strength, physical performance, BMI, ethnicity, comorbidities, medication use, and the modality used to quantify muscle mass was not reported. The case definition was based solely on low muscle mass and therefore differs from contemporary consensus definitions of sarcopenia. | 4 sarcopenia vs 4 normal muscle-biopsy samples. All 8 microgravity/gravity muscle-on-a-chip samples were excluded. | Public 2024 |
| **GSE111016** *Independent validation cohort* | Singapore Sarcopenia Study (SSS); Chinese community-dwelling older men; subseries of GSE111017/MEMOSA. All male. Age: controls 70.2 ± 4.1 years (65–79); sarcopenia 72.8 ± 4.2 years (66–78).  Migliavacca et al., Nature Communications 2019 (PMID: 31862890). | Homo sapiens; vastus lateralis muscle biopsy using a BioPince 16-G full-core needle; tissue was snap-frozen in liquid nitrogen and stored at −80 °C. | GPL16791; Illumina HiSeq 2500; total RNA-seq; TruSeq Stranded RNA Ribo-Zero Gold. RNA was isolated using QIAzol followed by miRNeasy purification. | 40 GEO samples: 20 healthy controls and 20 sarcopenia. The Asian Working Group criteria used in the source study required ALMI ≤7.00 kg/m² plus grip strength <26 kg and/or gait speed ≤0.8 m/s; muscle mass was measured by DXA and gait by a timed 6-m walk. **Potential confounders:** Chinese male-only cohort and a modest age shift toward older participants in the sarcopenia group. Although geographically and ethnically distinct, this validation set was generated within the same parent MEMOSA/GSE111017 project and laboratory framework as GSE111006 and GSE111010, and is therefore not fully independent at the study-generation level. | 20 sarcopenia vs 20 healthy controls; used exclusively for independent model validation in the current analysis. | Public 2019 |

Note: Ages are mean ± SD (range), recalculated from the GEO sample-level metadata for the samples included in the current analysis. ALMI, appendicular lean mass index; AWGS, Asian Working Group for Sarcopenia; BIA, bioelectrical impedance analysis; DXA, dual-energy X-ray absorptiometry; EWGSOP, European Working Group on Sarcopenia in Older People; NR, not reported. Diagnostic criteria, biopsy procedures, and cohort-level confounders were cross-checked against the original publications.
