# Supplementary material for: Integrative transcriptomic and experimental analyses prioritize TPT1 as a PANoptosis-associated candidate molecular marker in sarcopenia
Source: Front Cell Dev Biol. 2026 Aug 20;14:1912393. doi: 10.3389/fcell.2026.1912393 (PMC13490725; doi:10.3389/fcell.2026.1912393)
Supplement: Supplementary file 1 [file DataSheet1.zip › Supplementary_Table_S4.docx]

**Supplementary Table S4.** Primary antibodies used for western blotting

| **Antibody** | **Supplier** | **Working dilution** |
| --- | --- | --- |
| p53 | ABclonal | WB:1/500 |
| p16 | Proteintech | WB:1/500 |
| Myosin heavy chain (MyHC) | Solarbio | WB:1/500 |
| Myogenin | Solarbio | WB:1/1000 |
| MyoD | Proteintech | WB:1/1000 |
| TPT1/TCTP | Proteintech | WB:1/1000 |
| Atrogin-1/FBXO32 | ABclonal | WB:1/500 |
| MuRF-1/TRIM63 | Solarbio | WB:1/1000 |
| GAPDH | Proteintech | WB:1/4000 |
